# Longitudinal assessment of IFN-I activity and immune profile in critically ill COVID-19 patients with acute respiratory distress syndrome

**DOI:** 10.1186/s13054-021-03558-w

**Published:** 2021-04-12

**Authors:** Fabienne Venet, Martin Cour, Thomas Rimmelé, Sebastien Viel, Hodane Yonis, Remy Coudereau, Camille Amaz, Paul Abraham, Céline Monard, Jean-Sebastien Casalegno, Karen Brengel-Pesce, Anne-Claire Lukaszewicz, Laurent Argaud, Guillaume Monneret, the RICO study group, Remi Pescarmona, Lorna Garnier, Christine Lombard, Magali Perret, Marine Villard, Valérie Cheynet, Filippo Conti, Marie Groussaud, Marielle Buisson, Laetitia Itah, Inesse Boussaha, Françoise Poitevin-Later, Christophe Malcus, Morgane Gossez, Florent Wallet, Marie-Charlotte Delignette, Frederic Dailler, Marie Simon, Auguste Dargent, Pierre-Jean Bertrand, Neven Stevic, Marion Provent, Laurie Bignet, Valérie Cerro, Jean-Christophe Richard, Laurent Bitker, Mehdi Mezidi, Loredana Baboi

**Affiliations:** 1 Immunology Laboratory, Hôpital E. Herriot - Hospices Civils de Lyon, 5 place d’Arsonval, 69437 Lyon Cedex 03, France; 2 Joint Research Unit HCL-bioMérieux, EA 7426 “Pathophysiology of Injury-Induced Immunosuppression” (Université Claude Bernard Lyon 1 - Hospices Civils de Lyon - bioMérieux), 69003 Lyon, France; 3 Centre International de Recherche en Infectiologie (CIRI), Inserm U1111, CNRS, UMR5308, Ecole Normale Supérieure de Lyon, Université Claude, Bernard-Lyon 1, Lyon, France; 4 Medical Intensive Care Department, Edouard Herriot Hospital, Hospices Civils de Lyon, 69437 Lyon, France; 5 Anesthesia and Critical Care Medicine Department, Edouard Herriot Hospital, Hospices Civils de Lyon, 69437 Lyon, France; 6 Immunology Laboratory, Lyon-Sud University Hospital, Hospices Civils de Lyon, 69495 Pierre-Bénite, France; 7 Medical Intensive Care Department, Croix-Rousse University Hospital, Hospices Civils de Lyon, 69004 Lyon, France; 8 Centre d’Investigation Clinique de Lyon (CIC 1407 Inserm), Hospices Civils de Lyon, 69677 Lyon, France; 9 Virology Laboratory, Croix-Rousse University Hospital, Hospices Civils de Lyon, 69004 Lyon, France; 10 Immunology Laboratory, Lyon-Sud University Hospital, Hospices Civils de Lyon, Lyon, France; 11 Joint Research Unit HCL-bioMérieux, Lyon, France; 12 Centre d’Investigation Clinique de Lyon (CIC 1407 Inserm), Lyon, France; 13 Immunology Laboratory, Edouard Herriot Hospital, Hospices Civils de Lyon, Lyon, France; 14 RICO Clinical Investigators, Lyon, France; 15 Medical Intensive Care Department, Edouard Herriot Hospital, Hospices Civils de Lyon, Lyon, France; 16 Anesthesia and Critical Care Medicine Department, Edouard Herriot Hospital, Hospices Civils de Lyon, Lyon, France; 17 Medical Intensive Care Department, Croix-Rousse University Hospital, Hospices Civils de Lyon, Lyon, France

**Keywords:** COVID-19, ARDS, Type-I IFN, Immune profile, Immunosuppression

## Abstract

**Background:**

Since the onset of the pandemic, only few studies focused on longitudinal immune monitoring in critically ill COVID-19 patients with acute respiratory distress syndrome (ARDS) whereas their hospital stay may last for several weeks. Consequently, the question of whether immune parameters may drive or associate with delayed unfavorable outcome in these critically ill patients remains unsolved.

**Methods:**

We present a dynamic description of immuno-inflammatory derangements in 64 critically ill COVID-19 patients including plasma IFNα2 levels and IFN-stimulated genes (ISG) score measurements.

**Results:**

ARDS patients presented with persistently decreased lymphocyte count and mHLA-DR expression and increased cytokine levels. Type-I IFN response was initially induced with elevation of IFNα2 levels and ISG score followed by a rapid decrease over time. Survivors and non-survivors presented with apparent common immune responses over the first 3 weeks after ICU admission mixing gradual return to normal values of cellular markers and progressive decrease of cytokines levels including IFNα2. Only plasma TNF-α presented with a slow increase over time and higher values in non-survivors compared with survivors. This paralleled with an extremely high occurrence of secondary infections in COVID-19 patients with ARDS.

**Conclusions:**

Occurrence of ARDS in response to SARS-CoV2 infection appears to be strongly associated with the intensity of immune alterations upon ICU admission of COVID-19 patients. In these critically ill patients, immune profile presents with similarities with the delayed step of immunosuppression described in bacterial sepsis.

**Supplementary Information:**

The online version contains supplementary material available at 10.1186/s13054-021-03558-w.

## Introduction

The severe acute respiratory coronavirus-2 (SARS‐CoV‐2) is responsible for coronavirus disease-19 (COVID‐19) that mostly associates with asymptomatic and mild presentations but may progress in worst cases to severe pneumonia leading to intensive care unit (ICU) admission and acute respiratory distress syndrome (ARDS) requiring respiratory support [1]. Since the onset of the pandemic, the number of COVID-19 patients has never ceased to rise and has ultimately outstripped hospital/ICU capacity in some areas particularly affected by virus spread. COVID-19 mortality, although currently decreasing, has remained dramatically high especially in patients requiring invasive mechanical ventilation [1].

An enormous research effort has been consented to describe and understand the mechanisms sustaining altered host immune response to a virus totally unknown of human immune surveillance. Many exploratory non-hypothesis‐driven programs have been deployed in order to decipher immune processes at play in COVID‐19. Through various flow cytometry approaches, transcriptomic strategies, functional testing and multiplex measurement of soluble mediators, most studies compared immune response between groups of COVID-19 patients with increasing severity, i.e., mild/severe/critical [2–5]. These works provided homogenous results describing that, upon hospital arrival, the most severe phenotype associated with inflammatory response (e.g., moderate plasma IL-6 elevation) and altered cellular immunity, i.e., decreased monocyte HLA-DR expression (mHLA-DR) and marked lymphopenia [6–11]. In addition, impaired type I interferons (IFN-I) activity has emerged as a contributor to the disease severity [12–15]. These latter cytokines are crucial components of innate immune response against viruses by ringing a “first alarm” bell. IFN-I have, by themselves, antiviral properties but they also induce the expression of hundreds of IFN-stimulated genes (ISG) inducing cellular antiviral activity, therefore, limiting virus spread [5].

In contrast, fewer studies focused on longitudinal immune monitoring in hospitalized COVID-19 patients, whereas their hospital stay may last for several weeks [1]. This is especially true in critically ill COVID-19 patients with ARDS who also present with the highest mortality [1]. To investigate this particular aspect of COVID-19 immune response, we monitored selected immunological parameters, including IFNα2 measurement and IFN-stimulated genes (ISG) transcriptomic signature, in a group of 64 COVID-19 patients requiring ICU care over a 3-week period after ICU admission.

## Methods

### Clinical study design, patient population and approval

Between March and May 2020, critically ill patients admitted to three ICUs from academic hospital (Hospices Civils de Lyon, Lyon, France) who presented with pulmonary infection with SARS-CoV-2 confirmed by RT-PCR testing were prospectively included in the study. A flowchart describing the patient datasets used for the different analyses is provided in Additional file 1: Figure S1. Preliminary results from a subgroup of the cohort were published previously [9]. This project was part of an ongoing prospective observational clinical study (RICO, REA-IMMUNO-COVID). It was approved by ethics committee (Comité de Protection des Personnes Ile de France 1 - N°IRB/IORG #: IORG0009918) under agreement number 2020-A01079-30. This clinical study was registered at ClinicalTrials.gov (NCT04392401). The committee waived the need for written informed consent because the study was observational, with a low risk to patients, and no specific procedure, other than routine blood sampling, was required. Oral information and non-opposition to inclusion in the study were mandatory and were systematically obtained before any blood sample was drawn. This was recorded in patients’ clinical files. If a patient was unable to consent directly, non-opposition was obtained from the patient’s legally authorized representative and reconfirmed from the patient at the earliest opportunity. Inclusion criteria were: patients aged > 18 years, diagnosis of COVID-19 confirmed by RT-PCR testing in one respiratory sample. Inclusion criteria were (1) man or woman aged 18 or over, (2) hospitalization in ICU for SARS-CoV-2 pneumopathy, (3) first hospitalization in ICU, (4) positive diagnosis of SARS-CoV2 infection carried out by PCR or by another approved method in at least one respiratory sample, (5) sampling in the first 24 h after admission to ICU (D0) feasible and (6) patient or next of kin who has been informed of the terms of the study and has not objected to participating. Exclusion criteria were pregnancy, institutionalized patients, inability to obtain informed consent.

### Patient characteristics

For each patient, demographics, comorbidities, time from onset of COVID-19 symptoms to ICU admission, initial presentation of the disease in ICU including the ratio of the arterial partial pressure of oxygen to the fractional inspired oxygen (PaO_2_/FiO_2_ ratio) at admission, antiviral therapy targeting SARS-CoV-2 and organ support were documented. Organ dysfunctions according to sequential organ failure assessment (SOFA) score (range 0–24, with higher scores indicating more severe organ failures) and simplified acute physiology score II (SAPS II; range 0–164, with higher scores indicating greater severity of illness) were documented. Patients were classified in the acute respiratory distress syndrome (ARDS) group if they were invasively ventilated and met the Berlin criteria for ARDS within the first 3 days after ICU admission [16]. Follow-up included ICU length of stay, in-hospital mortality, day-28 (D28) mortality, day-90 (D90) mortality, as well as occurrence secondary infection based on recommendation from Comité technique des infections nosocomiales et des infections liées aux soins [17].

### Blood samples

Ethylene diamine tetraacetic acid (EDTA-)anticoagulated blood was drawn five times during the first month after ICU admission: within the first 48 h after admission (Day 0: D0), between 72 and 96 h after admission (D3), between D7 and D9 (D7), between D12 and D15 (D12) and between D20 and D25 (D20). Blood was stored at 4–8 °C and processed within 4 h after withdrawal. The numbers of available values for each immune parameter at each time point are presented in Additional file 1: Table S4.

### Cytokine measurement

Whole blood was sampled on EDTA tubes and plasma was frozen at − 20 °C within 4 h following blood collection. Cytokine measurement was taken by batches after 1 freeze/thaw cycle using standardized protocols fulfilling clinical and diagnostic laboratories accreditation requirements from the International Organization for Standardization. Plasma concentrations of IL-6, TNF-α, IFN-γ and IL-10 were measured by Simpleplex® technology using ELLA instrument (ProteinSimple®, San Jose, CA), following manufacturer’s instructions. Plasma IFNα2 concentrations were determined by single-molecule Array (SIMOA®) on a HD-1 Analyzer (Quanterix) using a commercial kit for IFN-α2 quantification (Quanterix, Lexington, Mass).

### IFN-stimulated genes (ISG) score calculation

Whole blood was collected on PAXgene blood RNA tubes (BD, Grenoble, France) for IFN signature and frozen at − 80 °C until RNA extraction. IFN score was obtained using nCounter® analysis technology (NanoString Technologies, Seattle, WA) by calculating the median of the normalized count of 6 ISGs using standardized protocols fulfilling clinical and diagnostic laboratories accreditation requirements from the International Organization for Standardization. As previously described, six interferon responsive genes were monitored: *SIGLEC1* (sialic acid binding Ig like lectin 1), *IFI27* (interferon alpha inducible protein27), *IFI44L* (interferon induced protein 44 like), *IFIT1* (interferon induced protein with tetratricopeptide repeats 1), *ISG15* (interferon-stimulated gene 15) and *RSAD2* (radical S-adenosyl methionine domain-containing 2). Three references genes were also measured: *ACTB* (Actin beta), *HPRT1* (hypoxanthine phosphoribosyltransferase 1) and *POLR2A* (RNA Polymerase II Subunit A) [18].

### Flow cytometry

T lymphocyte subpopulation immunophenotyping was performed on an automated volumetric flow cytometer from Beckman Coulter (Aquios CL) as previously described [19]. Monocyte HLA-DR expression and B and NK immunophenotyping were performed using antibodies from Beckman-Coulter and BD Biosciences. The expression of monocyte HLA-DR was determined using the Anti-HLA-DR/Anti-Monocyte Quantibrite assay (BD Biosciences, San Jose, USA). A total number of antibodies bound per cell (AB/C) were quantified using calibration with a standard curve determined with BD Quantibrite phycoerythrin (PE) beads (BD Biosciences) as described elsewhere [20]. B and NK lymphocyte immunophenotyping was performed using lyophilized antibody panel from Beckman Coulter (Duraclone kit). Data were acquired on a Navios flow cytometer (Beckman Coulter, Hialeah, FL), and flow data were analyzed using Navios software (Beckman Coulter). Enumeration of lymphocyte subpopulations as well as mHLA-DR measurement were performed using standardized protocols fulfilling clinical and diagnostic laboratories accreditation requirements from the International Organization for Standardization.

### SARS-CoV-2 detection by semiquantitative PCR

Semiquantitative values of SARS-CoV-2 viral load in upper respiratory samples at ICU admission were retrieved from clinical files for 40 patients. These results were obtained from accredited reference laboratory using RT-PCR technique with validated commercial kits (COBAS® SARS-CoV-2, Roche Diagnostics or reference technique from Pasteur Institute) based on recommendations from the Societé Française de Microbiology after evaluation of analytical performances of the techniques [21]. Patients were classified into three groups according to SARS-CoV-2 PCR Ct values reflecting respiratory viral load at admission (1) A significant viral excretion (ct value ≤ 33) subsequently divided into two groups: high viral load (Reference Ct value < 27, *n* = 12) and medium viral load (Reference Ct value = [27–33], *n* = 21); (2) a nonsignificant viral excretion corresponding to a low viral load (Reference Ct value = [33–37], *n* = 7).

### Statistical analysis

Data are presented as numbers and percentage (qualitative variables) and medians and 25th/75th percentiles (quantitative variables). Chi square or Fisher’s exact test were used for qualitative variables assessment. Quantitative variables were compared with Mann–Whitney U test. For all pairs of immune parameters, Spearman’s rho correlation coefficients were estimated and summarized in a correlation matrix. Kaplan–Meier survival curves were calculated in groups of patients with or without ARDS. The p-value of the log-rank test is given. The level of significance was set at 5%. Data were analyzed using Graphpad Prism version 5.03 (Graphpad Software, La Jolla, USA).

## Results

### Results on admission

Sixty-four patients with confirmed pulmonary SARS-CoV-2 infection admitted to three ICUs of Lyon University Hospitals (Hospices Civils de Lyon, Lyon, France) were included between March 16 and May 15, 2020. This period corresponded to the first surge in COVID-19 cases in France. Clinical characteristics are presented in Table 1. Median duration of symptoms before ICU admission was 7 [4–11] days. Forty patients (63%) presented with ARDS requiring mechanical ventilation within the first 3 days after ICU admission; 23/24 (96%) patients without ARDS needed noninvasive ventilation (including high flow nasal oxygenotherapy) for respiratory dysfunction/failure. Flowchart and the number of samples by time point are provided in Additional file 1: Figure S1A. As assessed after 28 days, mortality in this cohort was 22% with contrasting results between patients without ARDS (no mortality) and with ARDS (35%) (Additional file 1: Figure S1B).

**Table 1:** Clinical characteristics of critically ill patients with COVID-19 at ICU admission

	All patients (*n* = 64)	ARDS (*n* = 40)	No ARDS (*n* = 24)	*p* value
Demographics				
Age	65 [52–72]	66 [57–72]	55 [43–72]	0.0738
Gender	51 (80%)	36 (90%)	15 (63%)	**0.0116**
Body mass index (kg/m^2^)	28 [26–32]	29 [26–34]	28 [25–30]	0.2124
Body mass index > 30 kg/m^2^	23 (36%)	17 (43%)	6 (25%)	0.1874
Comorbidities				
Diabetes	16 (25%)	11 (25%)	5 (21%)	0.7693
Comorbidities				0.2979
0	36 (56%)	20 (50%)	16 (67%)	
≥ 1	28 (44%)	20 (50%)	8 (33%)	
Charlson score	0 [0–2]	1 [0–2]	0 [0–1]	**0.0002**
Admission symptoms				
Delay between first symptoms (Days)	7 [4–11]	7 [4–10]	8.5 [7–11]	0.2055
Fever	52 (81%)	34 (85%)	18 (75%)	0.3414
Cough	42 (66%)	24 (60%)	18 (75%)	0.2814
Dyspnea	40 (63%)	28 (70%)	12 (50%)	0.1208
Diarrhea	18 (28%)	10 (25%)	8 (33%)	0.5691
Diffuse pain	13 (20%)	7 (18%)	6 (25%)	0.5300
Altered general status	44 (69%)	24 (60%)	20 (83%)	0.0584
Other	28 (44%)	16 (40%)	12 (50%)	
Severity scores				
SOFA score	4 [2–8]	8 [4–9]	2 [2, 3]	**< 0.0001**
SAPS II score	34 [26–45]	40 [32–54]	27 [21–33]	**< 0.0001**
PaO_2_/FiO_2_ at admission	145 [92–191]	132 [95–166]	230 [83–298]	**0.0152**
Antiviral therapy				
Hydroxychloroquine	35 (55%)	20 (50%)	15 (63%)	0.4381
Lopinavir/ritonavir	5 (8%)	3 (8%)	2 (8%)	> 0.9999
Lopinavir/ritonavir + interferonβ	5 (8%)	4 (10%)	1 (4%)	> 0.9999
Remdesivir	1 (2%)	1 (3%)	0 (0%)	0.5238
Organ support				
Mechanical ventilation	63 (98%)	40 (100%)	23 (96%)	0.3750
Noninvasive ventilation	23 (36%)	0 (0%)	23 (100%)	< **0.0001**
Invasive ventilation	40 (63%)	40 (100%)	0 (0%)	** < 0.0001**
Vasoactive drugs	24 (38%)	24 (60%)	0 (0%)	** < 0.0001**
Renal replacement therapy	15 (23%)	15 (38%)	0 (0%)	**0.0004**
Follow-up				
Days in ICU	10 [4–30]	21 [11–58]	4 [2–7]	**0.003**
Days in hospital	21 [11–56]	33 [19–71]	13 [8–21]	**< 0.0001**
Hospital mortality	19 (30%)	19 (48%)	0 (0%)	**< 0.0001**
Day-28 mortality	14 (22%)	14 (35%)	0 (0%)	**< 0.0001**
Day-90 mortality	19 (30%)	19 (48%)	0 (0%)	**< 0.0001**
Secondary infections	32 (50%)	30 (75%)	2 (8%)	**< 0.0001**

The results are shown as medians and interquartile ranges [Q1–Q3] for continuous variables or numbers and percentage for categorical variables. Patients were separated into two groups based on presence of acute respiratory distress syndrome (ARDS) according to Berlin definition during the first 72 h after admission. Sepsis-related organ failure assessment (SOFA) and simplified acute physiology score II (SAPS II) scores were calculated during the first 24 h after intensive care unit (ICU) admission. Data were compared using nonparametric Mann–Whitney test for continuous variables or Fisher’s exact test for categorical variables

*p* values inferior to 0.05 are highlighted in bold

On admission, critically ill COVID-19 patients presented with elevated plasma levels of both pro- and anti-inflammatory cytokines (Additional file 1: Table S1). Marked lymphopenia (median = 653 cells/µL) affecting all lymphocyte subsets (e.g., median CD4 + T lymphocytes = 298 cells/µL, with CD4/CD8 ratio in normal range) [10, 11] and moderately decreased mHLA-DR (median = 11,125 AB/C, control values > 13,500 AB/C) [22] were also hallmarks of initial critically ill COVID-19 patients’ immune profile. Collectively, these results are in agreement with previously published values in ICU cohorts [9, 23, 24]. Interestingly, we observed that IFN-I response was induced in the majority of these patients as we measured elevated levels of plasma IFNα2 (median = 385 fg/mL, control values < 20 fg/mL) and ISG score (median = 40, control values < 2.3). These concentrations were consistent with the previously published results in cohorts of critically ill patients [12]. To note, for these inaugural immune parameters, no differences in their concentrations were observed based on delay between first symptoms and sampling time (data not shown).

**Fig. 1:**
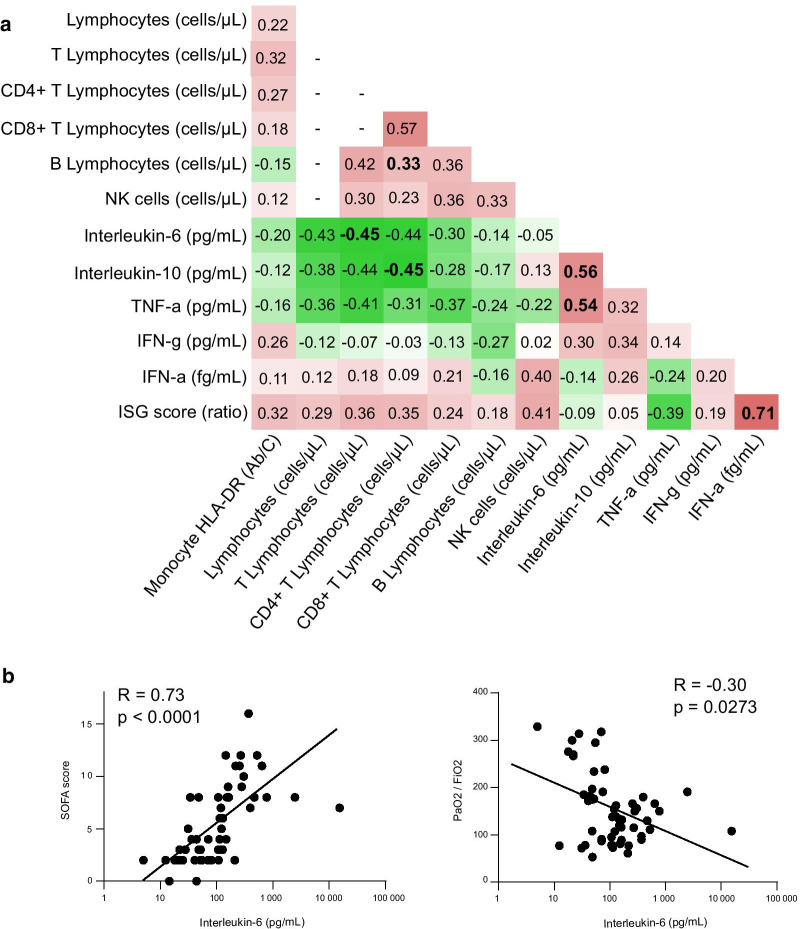
Correlation matrix of immune parameters at ICU admission. Immune parameters were measured at inclusion in 64 critically ill patients with COVID-19, and correlations were calculated using Spearman correlation tests. **a** Results are presented as a correlation matrix. Spearman correlation coefficients are plotted. Cells were colored according to the strength and trend of correlations (shades of red = positive, shades of green = negative correlations). Coefficients with a *p* value below 0.005 were highlighted in bold and considered significant after correction for multiple testing. Correlation results for non-independent parameters (i.e., lymphocyte subpopulations) are not presented. **b** Correlations between plasma interleukin-6 concentration at inclusion and sepsis-related organ failure (SOFA) score or PaO_2_/FiO_2_ ratio measured during first 24 h after admission are shown (*n* = 58). Corresponding logarithmic trendlines are shown

When correlating immune parameters at ICU admission (Fig. 1a), significant correlations were noted between increased pro-inflammatory IL-6 concentration and increased TNF-α and IL-10 levels (positive correlations), and decreased T lymphocyte number (negative correlation). No correlation was observed between IL-6 and IFNα2 levels or ISG score. In addition, initial increased IL-6 concentration was correlated with initial severity as measured by sepsis-related organ failure assessment (SOFA) score and with intensity of pulmonary dysfunction as measured by the ratio between partial pressure of oxygen in arterial blood (PaO_2_) and fraction of inspired oxygen (FiO_2_) (PaO_2_/FiO_2_ ratio, Fig. 1b). Finally, when classifying patients into three groups according to semiquantitative levels of viremia measured in upper respiratory samples, the group of patients with the highest viral load presented not only with higher plasma IL-6 concentrations at admission but also with a higher SOFA score and increased mortality (33%, Additional file 1: Figure S2). This suggests that the immune response is positively associated with viral burden and that failure to resolve both aspects may underlie severity [8].

### Comparison between ARDS and non-ARDS patients

**Fig. 2:**
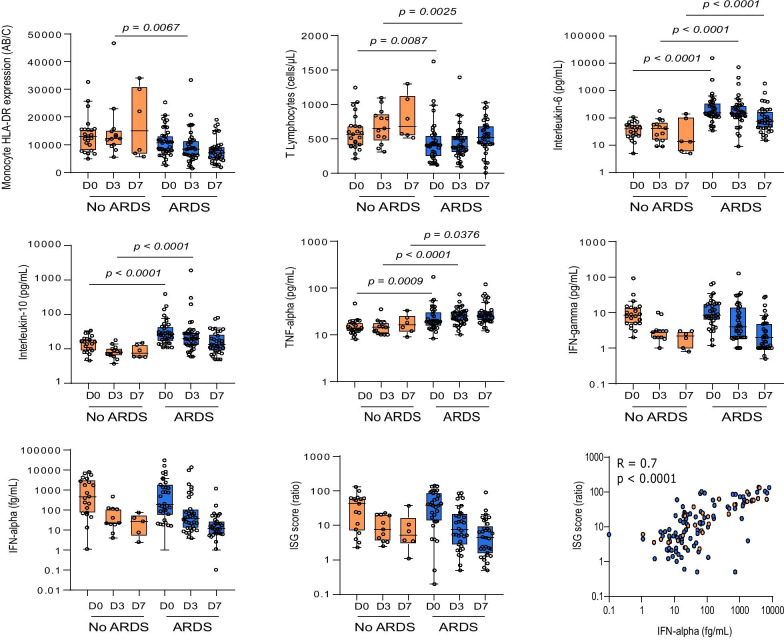
Immune response in critically ill COVID-19 patients with or without ARDS. Immune parameters were measured three times (D0: within the first 48 h, D3: between day 3 and day 4, D7: between D7 and D9) during the first week after ICU admission in COVID-19 patients. Patients were split in two groups based on presence (*n* = 40) or not (*n* = 24) of acute respiratory distress syndrome (ARDS) during the first 72 h after admission according to Berlin definition. Over time evolution of immune parameters including plasma IFNα2 concentration and type I interferon mRNA signature (ISG score) during the first week after admission in patients with (blue boxes and circles) or without ARDS (orange boxes and circles) is shown. In one ARDS patient at D0 and in one ARDS patient at D7, plasma IFNα2 concentrations were null and could not be plotted. Correlation between plasma IFNα2 concentrations and type I interferon signatures is presented. Two values could not be plotted in the group of ARDS patients because measured IFNα2 concentrations were null. Data are presented as Tukey box-plots and individual values. Nonparametric Mann–Whitney test was used to compare values between groups at the same time point. Spearman correlation test was used. Spearman correlation coefficient is shown. Only *p* values below 0.05 are shown. The results were not adjusted for multiple test comparisons

Considering the significant difference between ARDS and non-ARDS groups regarding D28 mortality (Additional file 1: Figure S1B), we then compared immune profiles between these 2 groups of patients (Fig. 2, Additional file 1: Table S2). As most non-ARDS patients presented with a short ICU stay of less than 1 week, we focused on the first three time points after ICU admission. Non-ARDS patients presented with a different immune profile than patients with ARDS, including higher lymphocytes count and mHLA-DR expression and lower cytokine levels and severity scores. Except for IL-6 and TNF-α, these differences persisted over the first week of follow-up (Fig. 2). We observed no difference between ARDS and non-ARDS patients regarding plasma IFNα2 measurements or ISG score (Fig. 2). The correlation between these two parameters was statistically significant (*r* = 0.7, *p* < 0.0001). In addition, we observed a rapid decrease of IFN-I markers after ICU admission in both groups. This shows that IFN-I response was induced in critically ill COVID-19 patients and efficient in activating downstream genes. However, the intensity of this response was not impacted by the development of pulmonary dysfunction or disease severity upon ICU admission.

### Comparison between survivors and non-survivors among ARDS patients

**Fig. 3:**
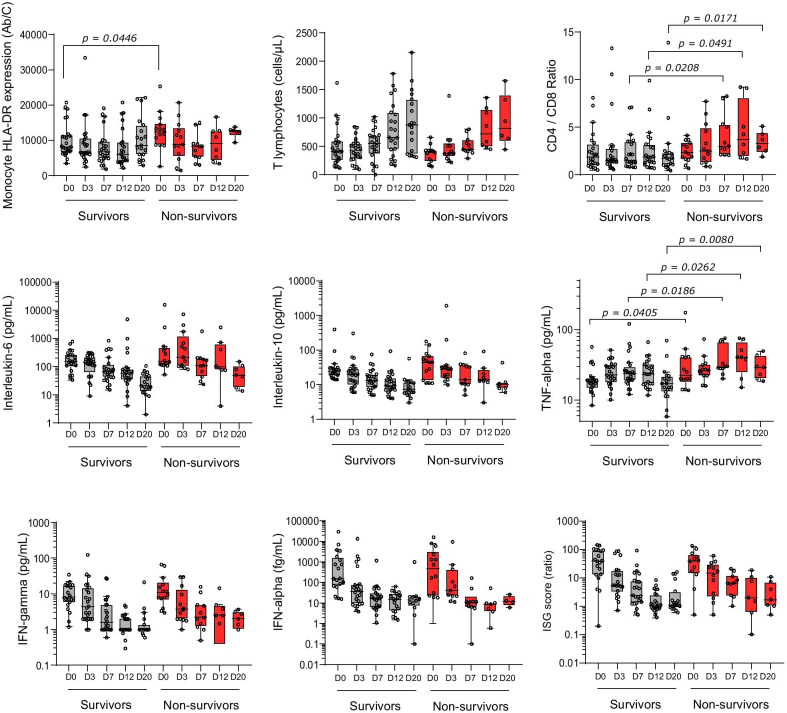
Immune response in COVID-19 ARDS patients according to status at D28. Immune parameters were measured 5 times (D0: within the first 48 h, D3: between day3 and D4, D7: between D7 and D9, D12: between D12 and D15, D20: between D20 and D25) during the first month after ICU admission in COVID-19 patients with ARDS. Patients were stratified in two groups according to their status at D28: survivors (*n* = 26) or non-survivors (*n* = 14). Over time evolution of immune parameters during the first month after admission in survivors (grey boxes) and non-survivors (red boxes) is shown. Regarding plasma IFNα2 level, in one non-survivor patient at D0 and in one survivor patient at D7, plasma IFNα2 concentration were null and could not be plotted. Data are presented as Tukey box-plots and individual values. Nonparametric Mann–Whitney test was used to compare values between groups at the same time point. Only *p* values below 0.05 are shown. The results were not adjusted for multiple test comparisons

We next focused on ARDS patients who presented with a prolonged ICU length of stay to evaluate immune profiles in regard with D28 mortality during a 3-week follow-up (Fig. 3, Additional file 1: Table S3). Clinical characteristics of survivors and non-survivors are presented in Table 2. Surprisingly, we did not observe any difference between survivors and non-survivors regarding the majority of immune parameters. In this cohort, survivors and non-survivors presented with apparent common immune responses mixing gradual return to normal ranges of cellular markers (mHLA-DR, lymphocyte count) and progressive decrease of cytokines levels including IFNα2 (and related ISG score). Only plasma TNF-α presented with a specific kinetic as it was the only cytokine to slowly rise during the follow-up. Moreover, its levels were significantly higher in non-surviving patients compared with survivors at all time points. This may reflect the high rates of secondary infections occurring in ARDS COVID-19 patients [25–27]. For example, in this cohort, 81% of survivors and 64% of non-survivors ARDS patients developed a secondary infection during their ICU stay. The constant elevation of neutrophil count during the whole monitoring in ARDS patients probably also reflect such re-stimulation of the immune system in response to secondary infectious challenges (Additional file 1: Table S3). Last, we observed that non-survivors exhibited a significant rise in CD4/CD8 ratio compared with survivors (Fig. 3). This was mainly due to the slower recovery of CD8+T cell numbers in this group (Additional file 1: Table S3).

**Table 2:** Clinical characteristics of patients with COVID-19 with ARDS according to status at D28

	Survivors (*n* = 26)	Non-survivors (*n* = 14)	*p* value
Demographics			
Age	65 [55–70]	67 [58–78]	0.3076
Gender	23 (88%)	13 (93%)	> 0.9999
Body mass index (kg/m^2^)	30 [27–35]	28 [24–30]	0.0960
Body mass index > 30 kg/m^2^	14 (54%)	3 (21%)	0.0921
Delay between first symptoms (Days)	7 [4–12]	5 [4–9]	0.1909
Comorbidities			
Comorbidities			0.3202
0	15 (58%)	5 (36%)	
≥ 1	11 (42%)	9 (64%)	
Charlson score	0 [0–2]	2 [0–2]	0.1281
Severity scores			
SOFA score	8 [3–10]	8 [4–8]	0.7937
SAPS II score	41 [31–52]	40 [33–59]	0.5893
PaO_2_/FiO_2_ at admission	150 [94–169]	116 [94–162]	0.2993
ARDS severity mild	1 (4%)	1 (7%)	> 0.9999
Moderate	18 (69%)	9 (64%)	> 0.9999
Severe	7 (27%)	4 (29%)	> 0.9999
Follow-up			
Days in ICU	40 [16–76]	11 [6–20]	**0.0008**
Days in hospital	64 [38–77]	15 [7–20]	**< 0.0001**
Secondary infections	21 (81%)	9 (64%)	0.2777

The results are shown as medians and interquartile ranges [Q1–Q3] for continuous variables or numbers and percentage for categorical variables. COVID-19 patients with ARDS were separated in two groups based on status at D28 after admission. Sepsis-related organ failure assessment (SOFA) and simplified acute physiology score II (SAPS II) scores were calculated during the first 24 h after admission. Patients were classified in ARDS severity groups according to Berlin criteria. ICU: intensive care unit. Data were compared using nonparametric Mann–Whitney test for continuous variables or Fisher’s exact test for categorical variables

*p* values inferior to 0.05 are highlighted in bold

## Discussion

Collectively, the present results provide an unbiased description of COVID-19 immuno-inflammatory derangements in critically ill COVID-19 patients focusing on ARDS patients who exhibit the highest mortality. We noted that, upon ICU admission, immune response to SARS-CoV-2 infection presents with similarities with bacterial sepsis [28, 29]. These include (1) severe lymphopenia affecting all lymphocyte subsets, (2) moderately decreased mHLA-DR and (3) moderately increased plasma cytokine levels showing at the same time both inflammatory (IL-6) and immunosuppressive (IL-10) responses. In addition, we noticed increased plasma IFNα2 levels and ISG score indicating the occurrence of an IFN-I response. This agrees with increased CD169 expression on monocytes (aka siglec-1, one of the six genes of ISG) in COVID-19 patients upon ICU admission [30]. Thus, the present results do not support a potentially altered IFN-I response in the majority of COVID-19 patients upon ICU admission. However, as IFNα2 concentrations reported elsewhere were higher in less severe/moderate patients (between 1000 and 5000 fg/mL) [12]; we cannot exclude that the incapacity to mount a full type-I IFN response immediately following SARS-CoV2 infection in some patients may have led to their worsening and ultimately to ICU admission.

These abnormalities (along with decreased plasmacytoid cells [12, 14, 31]) are reminiscent of the process of age-acquired immunosuppression (also called immunosenescence) observed in elderly people who are, by far, the primary victims of COVID-19. We may hypothesize that evolution of COVID-19 toward increasing severity in this population of old patients is mainly a consequence of this altered immune status [32–35]. For example, previous studies showed the negative correlation between lymphocytes count and pulmonary viral load [36, 37] while, elsewhere, the magnitude of pulmonary viral load was repeatedly associated with increased mortality [8, 38–43]. In the present work, this is also illustrated by the association between nasopharyngeal viral load and increased mortality, SOFA and IL-6 levels upon admission.

Our results emphasize that ARDS occurrence appears to be an important driver of mortality during COVID-19 progression in ICU patients [1]. In the present cohort, mortality occurred only in the group of patients with ARDS while all ICU patients without ARDS were released alive from the ICU. This observation makes a strong priority for avoiding progression toward ARDS in COVID-19 patients. After ICU admission, inflammatory response as well as IFN-I activity progressively normalized while immunological cellular parameters remained below references ranges. Thus, as seen in bacterial sepsis, ARDS occurrence in COVID-19 patients may secondarily amplify COVID-19-induced immune alterations either by direct cytotoxic effect and/or by negative anti-inflammatory feedbacks [28, 29, 32]. This leads to the development of a torpid immunosuppressed status in ARDS patients which may last several weeks [44].

In such critically ill patients with prolonged ICU stays, this immunosuppressed status presents with deleterious consequences. First, it probably participates in the long duration necessary to eradicate SARS-CoV-2 from the lung in invasively ventilated patients as described elsewhere [43]. For example, it was shown that the viral shedding in lower respiratory tract lasted almost 30 days in median in critically ill COVID-19 patients [38, 42]. Second, it most likely contributes to the very high rates of nosocomial infections reported not only in the present study (50%) but also in many others [26, 27, 45]. In particular, COVID-19 is characterized by astonishing elevated rates of secondary aspergillosis [46–48], a fungal disease usually seen in the most immunosuppressed patients. Such secondary infectious events may explain the persistently elevated TNF-α levels accompanied by increased neutrophil count in COVID-19 patients with ARDS and in non-survivors [25]. This suggests that in this second step of the disease, i.e., once ARDS occurred, immunostimulation could represent a sound approach to try to promote immune recovery and prevent secondary bacterial and fungal infections.

The most striking result of the present study was to observe similar altered immune response in both surviving and non-surviving ARDS patients over a 3-week follow-up. Thus, the duration of this COVID-19 induced immunosuppressed status remains to be defined. While a recent study observed that the onset of T cell recovery in COVID-19 ICU patients with ARDS started on day 35 [49], the 3-week ICU follow-up performed in this study may have been too short to distinguish immune trajectories according to outcomes in COVID-19 ARDS patients. Of note, in this latter work [49], patients with unfavorable outcome presented with increased CD4/CD8 ratio as observed in the present study. We may thus hypothesize that, in ARDS patients, the lack of CD8+T lymphocyte recovery could be a poor prognosis factor [35, 50].

Being exploratory, our work presents with limitations. First, SARS-CoV2 viral load could not be regularly monitored. Further studies should include strict quantitative evaluation of viral load with standardized tools throughout the monitoring to decipher the duration of viral persistence in the lung and its correlation with immune response and outcomes. Second, functional testing of immune cells was not performed. If available, this should be incorporated in addition with phenotypic markers of immune response [51–53]. This is especially true regarding CD8+T cell functionality in response to SARS-CoV-2 peptides that may help to understand which comes first: CD8 efficiency (and recovery) or virus disappearance. Three, we did not include less severe COVID-19 cohorts in order to explore (with single-molecule array—SIMOA-technology) the magnitude of IFN-I response in patients who correctly eradicated the virus. Last, at the time of first COVID-19 surge in France (corresponding to the patients reported here), optimized care protocols (oxygen, heparin, dexamethasone) were not applied. Therefore, some observations need to be confirmed under the angle of current clinical practice.

## Conclusions

In sum, upon patients’ admission to the ICU, pulmonary virus spread is accompanied by an inflammatory response characterized by moderately increased circulating levels of typical inflammatory cytokines (e.g., IL-6 levels usually < 100 pg/mL) [54–57]. At this stage, no obvious observation of altered IFN-I response could be reported. If not appropriately controlled by the immune system [33], virus replication in lungs and related inflammation may progressively lead to ARDS which appears to be one driver of mortality. Following this acute response leading to pulmonary dysfunction, inflammatory response rapidly declined. As observed in bacterial sepsis [28, 29], patients subsequently present with a marked delayed immunosuppression. This state of immunosuppression likely prevents efficient virus eradication from the lung, facilitates virus spread outside lungs as illustrated by the deleterious association of persistent viremia and mortality [58–62]. This also probably favors the occurrence of frequent secondary infections with opportunistic pathogens [26, 27, 47]. All these elements explain the long ICU stay of invasively ventilated COVID-19 patients. In the current 3-week monitoring of ARDS patients, we did not identify any immunological parameter that significantly associated with mortality. Thus, the better understanding of the mechanisms which finally permit survival after several weeks in ICU is a crucial issue for next studies.

## Supplementary Information

**Additional file 1. Table S1.** Immune response in critically ill COVID-19 patients, **Table S2.** Immune response in critically ill COVID-19 patients with or without ARDS, **Table S3.** Immune response in critically COVID-19 patients with ARDS according to status at D28, **Table S4.** Numbers of available values for each immune parameter at each time point; **Figure S1.** Flowchart and Survival Curves and **Figure S2.** Impact of viral load at admission.

## Data Availability

The datasets analyzed during the current study are available from the corresponding author on reasonable request.

## Abbreviations

AB/C: Numbers of antibodies bound per cell
ARDS: Acute respiratory distress syndrome
HLA-DR: Human leukocyte antigen-DR
ICU: Intensive care unit
ISG: Interferon-stimulated genes
SAPS II: Simplified acute physiology score II
SOFA: Sequential organ failure assessment score
PaO_2_/FiO_2_ ratio: Ratio of the arterial partial pressure of oxygen to the fractional inspired oxygen
