# Supplementary material for: Longitudinal assessment of IFN-I activity and immune profile in critically ill COVID-19 patients with acute respiratory distress syndrome
Source: Crit Care. 2021 Apr 12;25:140. doi: 10.1186/s13054-021-03558-w (PMC8040759; doi:10.1186/s13054-021-03558-w)

Supplementary information

Longitudinal assessment of IFN-I activity and immune profile in critically ill COVID-19 patients with acute respiratory distress syndrome

Fabienne Venet[1,2,3], Martin Cour[4], Thomas Rimmelé[2,5], Sebastien Viel[3,6], Hodane Yonis[7], Remy Coudereau[1,2], Camille Amaz[8], Paul Abraham[5], Céline Monard[5], Jean-Sebastien Casalegno[9], Karen Brengel-Pesce[2], Anne-Claire Lukaszewicz[2,5], Laurent Argaud[4], Guillaume Monneret[1,2] and the RICO study group

Supplemental Table 1. Immune response in critically ill COVID-19 patients. Immune parameters were measured 5 times (D0: within the first 48h, D3: between day3 and day 4, D7: between D7 and D9, D15: between D12 and D15, D20: between D20 and D25) after ICU admission in 64 COVID-19 patients. Results are presented as medians and interquartile ranges [Q1-Q3]. Numbers of available samples at each time-point are listed. ISG: Interferon stimulated genes. Ly: lymphocytes. References values are provided by routine clinical immunology laboratories at our institution.

	**D0 (n = 62)**	**D3 (n = 50)**	**D7 (n = 41)**	**D12 (n = 32)**	**D20 (n = 26)**	**Reference values**
**Ly count (cell/µL)**	653 [479 - 892]	702 [499 - 919]	750 [546 - 1014]	1017 [634 - 1485]	1236 [742 - 1709]	[1 000 – 2 800]
**CD4+ T Ly count (cell/µL)**	298 [186 - 362]	288 [189 - 405]	324 [251 - 508]	446 [266 - 701]	550 [272 - 976]	[336 - 1 126]
**CD8+ T Ly count (cell/µL)**	141 [84 - 201]	142 [78 - 218]	152 [88 - 272]	209 [113 - 352]	257 [151 - 424]	[125 - 780]
**CD4 / CD8 Ly Ratio**	2.0 [1.3 – 3.0]	1.9 [1.3 - 2.9]	2.2 [1.4 - 3.5]	2.2 [1.4 – 4.0]	2.1 [1.1 - 3.0]	[1 - 4.9]
**Monocyte HLA-DR (AB/C)**	11 125 [7 667 – 15 419]	9 121 [6 194 – 12 765]	7 347 [5 117 – 9 921]	6 914 [3 987 – 12 155]	10 838 [6 848 – 13 123]	[13 500 – 45 000]
**IL-6 (pg/mL)**	108 [44 - 213]	118 [45 - 217]	68 [32 - 155]	70 [38 - 105]	29 [16 - 56]	< 7 pg/mL
**IL-10 (pg/mL)**	20 [14 - 30]	15 [8 - 28]	13 [8 - 22]	12 [7 - 17]	8 [6 - 12]	< 8 pg/mL
**TNF-α (pg/mL)**	18 [13 - 20]	22 [15 - 30]	24 [19 - 32]	28 [17 - 40]	20 [15 - 26]	< 10 pg/mL
**IFN-γ (pg/mL)**	8 [5 - 16]	3 [2 - 10]	2 [1 - 5]	2 [1 - 3]	1 [1 - 3]	< 3 pg/mL
**IFN-α (fg/mL)**	385 [67 – 2 407]	35 [12 - 110]	13 [6 - 33]	10 [4 - 29]	13 [8 - 20]	< 20 fg/mL
**ISG score (Ratio)**	40 [12 - 68]	8 [3 - 20]	4 [2 - 11]	1 [1 - 3]	1 [1 - 4]	< 2.3

Supplemental Table 2. Immune response in critically ill COVID-19 patients with or without ARDS. Immune parameters were measured 3 times (D0: within the first 48h, D3: between day3 and day 4, D7: between D7 and D9) during the first week after ICU admission in COVID-19 patients. Patients were separated in two groups based on presence (n = 40) or not (n = 24) of acute respiratory distress syndrome (ARDS) during the first 72h after admission according to Berlin definition. Medians and interquartile ranges [Q1-Q3] are shown. Numbers of available data are listed. Because of the high number of missing values for this set of biological parameters, no statistical analyses were performed.

	No ARDS			ARDS		
	**D0**	**D3**	**D7**	**D0**	**D3**	**D7**
**Lactates (mM)**	1.2<br>[1 – 1.3]<br>n = 12	1.5<br>[1.3 – 1.6]<br>n = 3	NA	1.6<br>[1.3 – 2.2]<br>n = 34	1.8<br>[1.5 – 2.3]<br>n = 27	1.7<br>[1.5 – 2]<br>n = 22
**Leucocytes (G/L)**	7.7<br>[4.4 – 8.8]<br>n = 18	6.7<br>[5.8 – 8.3]<br>n = 7	6.9<br>[5.9 – 7.7]<br>n = 3	8.2<br>[6.8 – 9.6]<br>n = 29	9.5<br>[8.1 – 13.6]<br>n = 28	11.8<br>[9 – 13.7]<br>n = 24
**Neutrophils (G/L)**	5.2<br>[3.1 – 7.3]<br>n = 18	5.5<br>[3.9 – 5.9]<br>n = 7	4.2<br>[3.8 – 5.9]<br>n = 3	6.6<br>[4.4 – 8.3]<br>n = 29	9.2<br>[6.8 – 11.7]<br>n = 28	8.6<br>[6.8 – 11.5]<br>n = 24
**Monocytes (G/L)**	0.35<br>[0.29 – 0.47]<br>n = 17	0.6<br>[0.51 – 0.81]<br>n = 7	0.8<br>[0.39 – 0.83]<br>n = 3	0.33<br>[0.2 – 0.46]<br>n = 29	0.5<br>[0.34 – 0.79]<br>n = 28	0.9<br>[0.51 – 1.1]<br>n = 23
**CRP (g/L)**	119<br>[67 – 191]<br>n = 18	203<br>[114 – 295]<br>n = 4	20<br>[9 – 32]<br>n = 2	155<br>[103 – 262]<br>n = 22	221<br>[198 – 333]<br>n = 3	144<br>[51 – 181]<br>n = 3
**Lymphocytes (cells/µL)**	769<br>[609– 1047]<br>n = 22	941<br>[630 – 1292]<br>n = 12	996<br>[804 – 1495<br>n = 6	615<br>[424 – 856]<br>n = 39	559<br>[436 – 804]<br>n = 37	702<br>[532 – 995]<br>n = 35
**T CD4+ Lymphocytes (cells/µL)**	346<br>[256 – 431]<br>n = 23	399<br>[267 – 643]<br>n = 13	472<br>[313 – 920]<br>n = 6	287<br>[140 – 342]<br>n = 39	246<br>[176 – 365]<br>n = 37	315<br>[234 – 496]<br>n = 35
**T CD8+ Lymphocytes (cells/µL)**	178<br>[125 – 244]<br>n = 23	211<br>[134 – 235]<br>n = 13	217<br>[127 – 415]<br>n = 6	120<br>[73 – 189]<br>n = 38	133<br>[68 – 185]<br>n = 36	148<br>[79 – 254]<br>n = 34
**CD4/CD8 Ratio**	2<br>[1.4 – 2.4]<br>n = 23	2<br>[1.5 – 2.7]<br>n = 13	2.7<br>[1.7 – 4.8]<br>n = 6	2.1<br>[1.2 – 3.3]<br>n = 38	1.7<br>[1.3 – 3.1]<br>n = 36	2.1<br>[1.4 – 3.6]<br>n = 33
**B Lymphocytes (cells/µL)**	84<br>[78 – 120]<br>n = 17	149<br>[107– 165]<br>n = 5	165<br>[163 – 167]<br>n = 2	94<br>[52 – 147]<br>n = 32	84<br>[72 – 113]<br>n = 15	57<br>[47 – 105]<br>n = 6
**NK cells (cells/µL)**	84<br>[48 – 125]<br>n = 17	69<br>[47 – 96]<br>n = 5	85<br>[83 – 86]<br>n = 2	96<br>[54 – 134]<br>n = 32	59<br>[43 – 115]<br>n = 15	19<br>[9.5 – 46.5]<br>n = 5

Supplemental Table 3. Immune response in critically COVID-19 patients with ARDS according to status at D28. Immune parameters were measured 5 times (D0: within the first 48h, D3: between day3 and D4, D7: between D7 and D9, D15: between D12 and D15, D20: between D20 and D25) during the first month after ICU admission in COVID-19 patients with ARDS. Patients were stratified in two groups according to their status at D28: survivors (n = 26) or non-survivors (n = 14). Medians and interquartile ranges [Q1-Q3] are shown. Numbers of available data are listed. Non parametric Mann Whitney test was used to compare values between groups at the same time-point.* p < 0.05 and ** p < 0.005 versus corresponding value at the same time-point in survivors.

	**Survivors**					**Non-Survivors**				
	**D0**	**D3**	**D7**	**D15**	**D20**	**D0**	**D3**	**D7**	**D15**	**D20**
**Lactates (mM)**	1.5 [1.1 – 2.1] n = 21	1.7 [1.4 – 2.2] n = 17	1.7 [1.5 – 2.0] n = 16	1.5 [1.3 – 1.7] n = 11	1.8 [0.9 – 3.1] n = 5	2.0 [1.6 – 2.5] n = 13	1.9 [1.7 – 3.7] n = 10	2.0 [1.5 – 2.2] n = 6	2.0 [1.4 – 2.3] n = 6	1.6 [1.4 – 1.7] n = 2
**Leucocytes (G/L)**	8 [7 – 11] n = 18	11 [8 – 16] n = 19	12 [8 – 14] n = 17	13 [10 – 17] n = 20	14 [10 – 20] n = 14	8 [6 – 9] n = 11	9 [7 – 12] n = 9	12 * [9 – 16] n = 7	11 [7 – 14] n = 5	12 [11 – 20] n = 3
**Neutrophils (G/L)**	6.8 [4.3 – 8.2] n = 18	9.2 [6.6 – 12] n = 19	8.7 [6.4 – 12] n = 17	10 [7.8 – 13.3] n = 20	11.2 [7.7 – 17] n = 14	6.6 [4.3 – 8.4] n = 11	7.7 [6.9 – 11.0] n = 9	8.1 [6.9 – 10.9] n = 7	8.1 [5.4 – 9.2] n = 5	9.5 [9.3 – 12.9] n = 3
**Monocytes (G/L)**	0.4 [0.2 – 0.5] n = 18	0.5 [0.4 – 0.8] n = 19	0.9 [0.5 – 1.2] n = 16	1 [0.6 – 1.5] n = 20	1 [0.6 – 1.1] n = 14	0.3 [0.2 – 0.5] n = 11	0.5 [0.2 – 0.8] n = 9	0.9 [0.7 – 1.1] n = 7	0.4 [0.2 – 1.1] n = 5	0.9 [0.6 – 1.7] n = 3
**CRP (g/L)**	168 [106 – 278] n = 15	NA	NA	NA	NA	120 [59 – 211] n = 6	NA	NA	NA	NA
**Lymphocytes (cells/µL)**	623 [430– 898] n = 25	572 [405 – 852] n = 24	726 [482 – 1015] n = 24	988 [585 – 1510] n = 23	1141 [608 – 1737] n = 19	564 [375 – 849] n = 14	545 [503 – 790] n = 13	688 [532 – 980] n = 11	988 [732– 1624] n = 8	1214 [730– 1630] n = 6
**T CD4+ Lymphocytes (cells/µL)**	306 [153 – 356] n = 25	231 [167 – 344] n = 24	284 [189 – 485] n = 24	441 [237 – 627] n = 23	461 [203 – 730] n = 19	267 [117 – 309] n = 14	267 [176 – 427] n = 13	365 [302 – 503] n = 11	593 [344 – 888] n = 8	653 [449 – 1025] n = 6
**T CD8+ Lymphocytes (cells/µL)**	142 [67 – 212] n = 24	157 [58 – 224] n = 23	208 [84 – 287] n = 23	230 [111 – 370] n = 23	257 [156 – 427] n = 19	97 [75 – 143] n = 14	99 [69 – 159] n = 13	112 [65 – 144] n = 11	195 [95– 221] n = 8	173 [115 – 410] n = 6
**B Lymphocytes (cells/µL)**	107 [69 – 154] n = 18	87 [68– 108] n = 8	57 [51 – 118] n = 4	88 [47 – 102] n = 3	NA	80 [47 – 125] n = 14	80 [72 – 243] n = 7	66 [38 – 94] n = 2	NA	NA
**NK cells (cells/µL)**	100 [54 – 128] n = 18	61 [41 – 109] n = 8	16 [8 – 35] n = 4	40 [14 – 61] n = 3		89 [51– 160] n = 14	59 [43 – 271] n = 7	NA	NA	NA

Supplementary Table 4. Numbers of available values for each immune parameter at each time point. ARDS: acute respiratory distress syndrome. D: Day. mHLA-DR: monocytic expression of antigen-leukocyte DR. IL: interleukin. TNF: Tumor necrosis factor. IFN: interferon. ISG: interferon stimulated genes.

A- Numbers of values presented in Figure 2

	No ARDS			ARDS		
	D0	D3	D7	D0	D3	D7
mHLA-DR	23	13	6	39	37	35
T lymphocytes	23	13	6	39	37	35
IL-6	22	13	6	36	37	35
IL-10	22	13	6	36	37	35
TNF-α	22	13	6	36	37	35
IFN-γ	22	13	6	36	37	35
IFN-α2	21	11	5	34	35	33
ISG score	21	11	5	32	34	34

B- Numbers of values presented in Figure 3

	Survivors					Non-survivors				
	D0	D3	D7	D15	D20	D0	D3	D7	D15	D20
mHLA-DR	25	24	24	23	19	14	13	11	8	6
T lymphocytes	25	24	24	23	19	14	13	11	8	6
CD4/CD8 ratio	24	23	22	23	19	14	13	11	8	6
IL-6	22	24	24	22	19	14	13	11	8	6
IL-10	22	24	24	22	19	14	13	11	8	6
TNF-α	22	24	24	22	19	14	13	11	8	6
IFN-γ	22	24	24	22	19	14	13	11	8	6
IFN-α2	20	24	23	18	12	14	11	10	7	4
ISG score	20	22	23	22	19	12	12	10	7	6

Supplemental Figure 1. Flow Chart and Survival Curves.

A- Study flow diagram. Patients were split in two groups based on presence of acute respiratory distress syndrome (ARDS) according to Berlin definition during the first 72h after admission. Patients with ARDS were then analyzed according to the occurrence of deaths within 28 days after ICU admission. The numbers of available samples at each time-point in each subgroup of patients are shown. **B-** Kaplan-Meier survival curves over the first 28 days after admission are shown for patients with (dashed red line) or without ARDS (black line). 95 % confidence interval is shown for patients with ARDS. Log-rank test was used to compare survival between the two groups.

Supplemental Figure 1

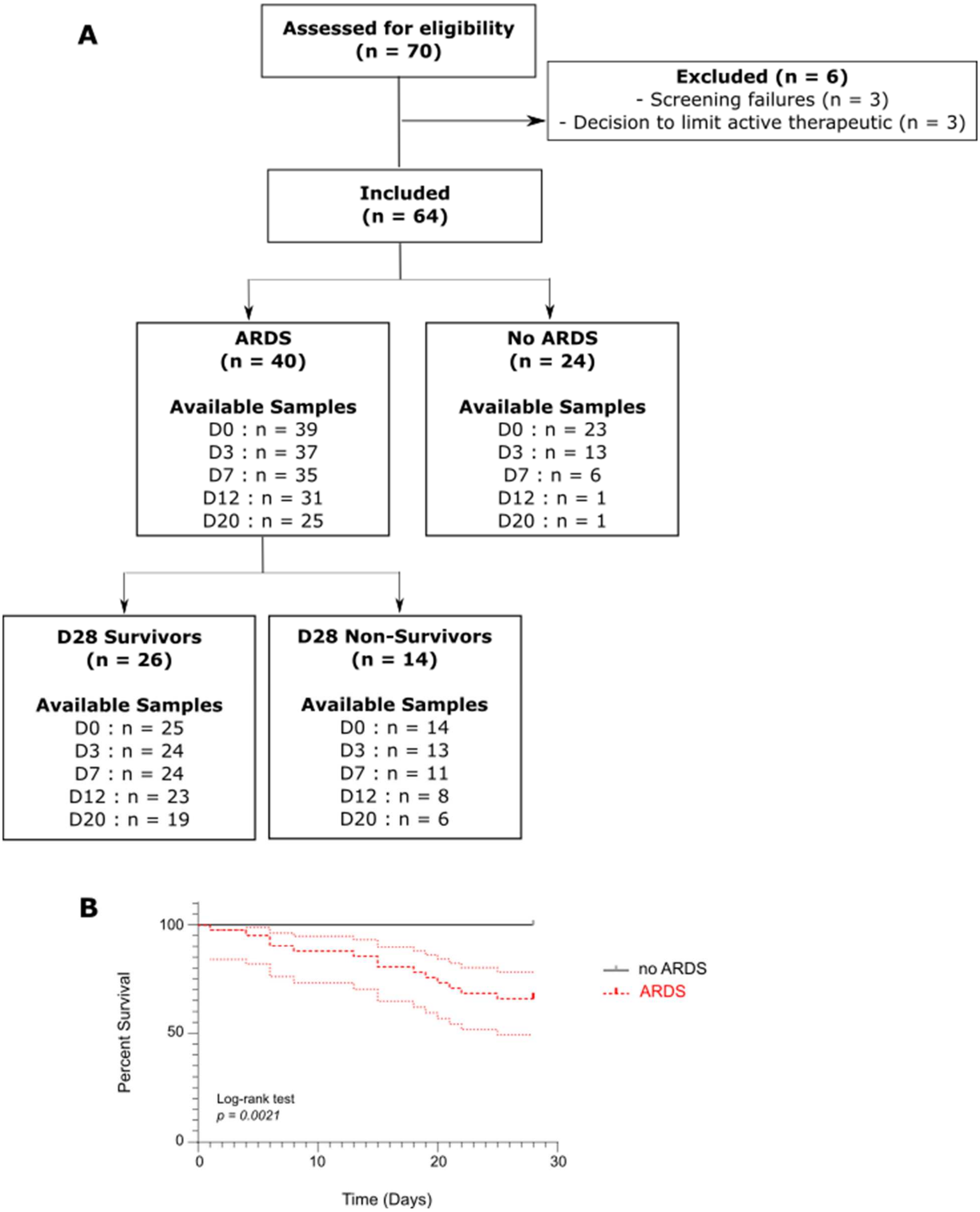

Supplemental Figure 2. Impact of viral load at admission.

Semi-quantitative values of SARS-CoV-2 viral load in upper respiratory samples at ICU admission were retrieved from clinical data files for 40 patients. Patients were classified in 3 groups according to SARS-CoV-2 PCR Ct values: High viral load (High, n = 12), Medium viral load (Med, n =21), Low viral load (Low, n =7). Plasma interleukin-6 and SOFA score at admission are shown in each group. Data are presented as individual values and Tukey box plots. Pie charts represent the percentages of survivors (grey parts) and non-survivors (red parts) in each group.

Supplemental Figure 2

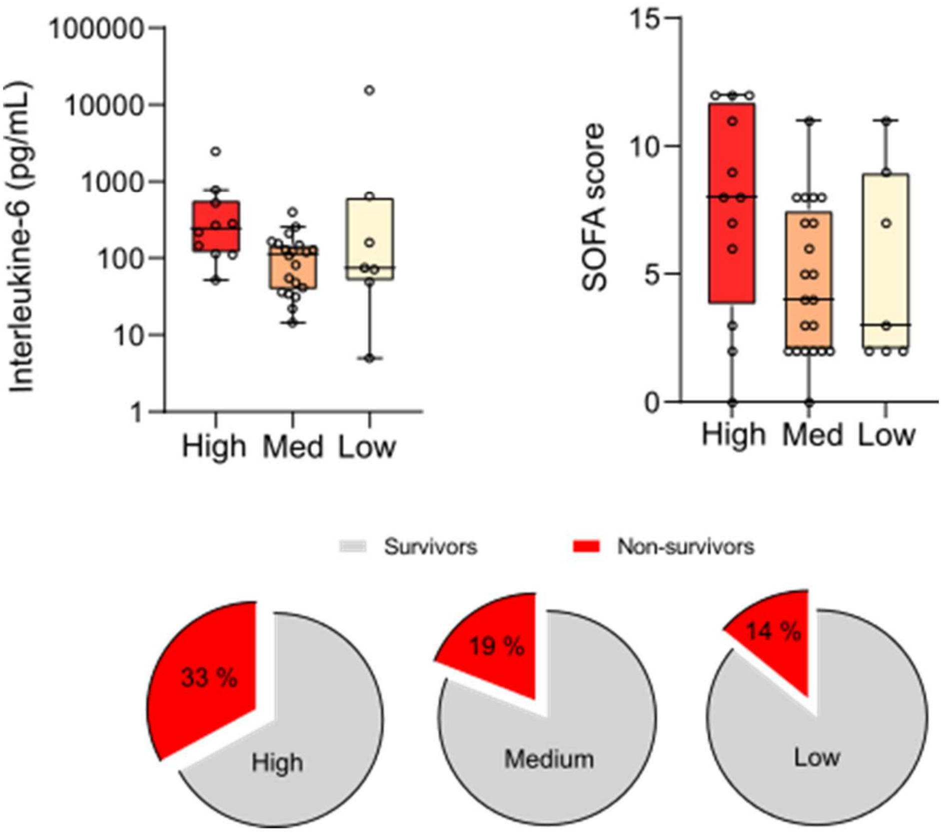

Supplement: Supplementary file 1 — Additional file 1. Table S1. Immune response in critically ill COVID-19 patients, Table S2. Immune response in critically ill COVID-19 patients with or without ARDS, Table S3. Immune response in critically COVID-19 patients with ARDS according to status at D28, Table S4. Numbers of available values for each immune parameter at each time point; Figure S1. Flowchart and Survival Curves and Figure S2. Impact of viral load at admission. [file 13054_2021_3558_MOESM1_ESM.pdf]
